# *Cirolana songkhla*, a new species of brackish-water cirolanid isopod (Crustacea, Isopoda, Cirolanidae) from the lower Gulf of Thailand

**DOI:** 10.3897/zookeys.375.6573

**Published:** 2014-01-30

**Authors:** Eknarin Rodcharoen, Niel L. Bruce, Pornsilp Pholpunthin

**Affiliations:** 1 Department of Biology, Faculty of Science, Prince of Songkla University, Hat Yai, Songkhla, Thailand 90112; 2 Museum of Tropical Queensland, Queensland Museum and School of Marine and Tropical Biology, James Cook University; 70–102 Flinders Street, Townsville, Australia 4810; Department of Zoology, University of Johannesburg, South Africa

**Keywords:** Isopoda, Cirolanidae, *Cirolana*, new species, brackish water, Thailand

## Abstract

*Cirolana songkhla* **sp. n.** was collected from brackish-water habitats including lagoons and estuaries in the coastal zone of the lower Gulf of Thailand. *C. songkhla* **sp. n.** is described and fully illustrated; *C. songkhla* **sp. n.** can be recognized by the presence of abundant chromatophores dorsally, lack of ornamentation on the posterior pereonites, pleonites and pleotelson, the number of robust setae on the uropodal and pleotelson margins (uropod exopod lateral margin with 12–14 RS, mesial margin with 5–8 RS; endopod lateral margin with 8–10 RS, mesial margin with 11–13 RS; pleotelson with 12–15 RS) and lack of setae on the endopods of pleopods 3–5. A dichotomous key of brackish *Cirolana* species in Thailand is given.

## Introduction

The family Cirolanidae has received little attention in Thailand and South-East Asia in general, with only 13 species in eight genera known from Thailand. The genus *Cirolana* Leach, 1818, the largest genus in the family with 129 species (Bruce and Schotte 2013) is equally poorly known in the region. Suvatti (1967) listed the known species from Thailand, while Kensley (2001) listed the species known to date from the Indian Ocean, including the western coasts of Thailand. Recently, Bruce and Olesen (2002) have reported four marine cirolanid species from Andaman Sea including two new species of *Cirolana*. All the brackish species of *Cirolana* have been recorded only from the Gulf of Thailand: *Cirolana willeyi* Stebbing, 1904 from the Mae Klong River, Samut Songkhram province (Upper Gulf of Thailand) (Suvatti 1967); and *Cirolana pleonastica* Stebbing, 1900 and *Cirolana parva* Hansen, 1890 from Songkhla Lake (Lower Gulf of Thailand) (Chilton 1924, 1926). Chilton’s (1924, 1926) records are both now regarded as misidentification as Barnard (1935) suggested that the record of *Cirolana pleonastica* was in fact *Cirolana fluviatilis* Stebbing, 1902 and Bruce and Bowman (1982) showed that *Cirolana parva* is restricted to the Caribbean and Central American coasts. Although there had been numerous relatively recent advances in the taxonomy of the Cirolanidae (e.g. see Bruce 1986, Brusca et al. 1995, Keable 2006, Moore and Brusca 2003, Riseman and Brusca 2002), knowledge on Thailand’s fauna remains minimal.

This present report corrects Chilton’s record of *Cirolana parva*, describes *Cirolana songkhla* sp. n., and presents a key of the brackish-water species of *Cirolana* that occur in Thailand.

## Materials and Methods

**Figure 1.**
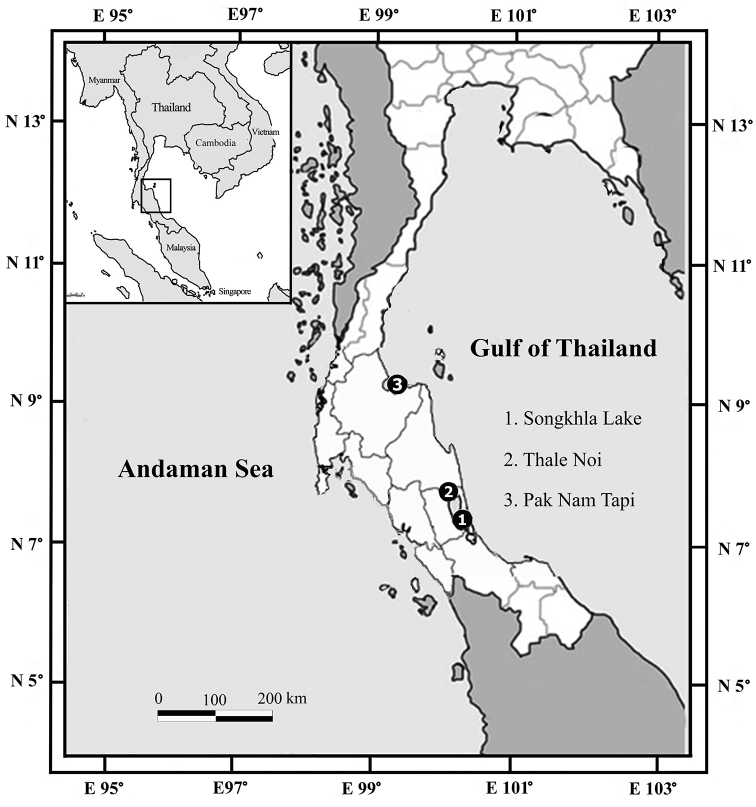
Map of sampling sites.

Abbreviations: PMS, plumose marginal setae; RS, robust seta/setae; CPS, circumplumose setae.

Specimens were collected by using baited traps from brackish-water habitats including the lagoon and estuary in the lower Gulf of Thailand (Figure 1). Appendages were dissected and drawn under stereo and compound microscopes with a camera lucida. Morphological characters for the description (based on Bruce 2004) were prepared by using DELTA (Descriptive Language for Taxonomy: Dallwitz et al. 1997). The type material of the new species is deposited at Prince of Songkla University Zoological Collection (PSUZC) and Museum of Tropical Queensland (MTQ).

## Taxonomy

### Genus Cirolana

Leach, 1818

http://species-id.net/wiki/Cirolana

#### Restricted synonymy.

Bruce 1986: 139, Kensley and Schotte 1989: 132, Brusca et al. 1995: 17.

#### Remarks.

*Cirolana* is the largest genus of the Cirolanidae (Bruce 1981, 1986, Brusca et al. 1995, Keable 2006) with 129 named species (Bruce and Schotte 2013) and many more not yet described. *Cirolana* occurs from cool-temperate to tropical regions, primarily in marine environments, but also occasionally found in low-salinity habitats, such as mangroves, estuarine reaches of rivers and creeks (Bruce 1986), and also rarely found in freshwater and cave and ground waters (Kensley and Schotte 1989, Botosaneanu and Iliffe 2000). Most low salinity and freshwater species of *Cirolana* lack setae on the endopods of pleopods 3 and 4, and were formerly placed in the genus *Anopsilana* Paulian & Delamare-Debouteville, 1956 (e.g. Bruce 1986; Bruce and Iliffe 1993), but following Botosaneanu and Iliffe (1997) the genus is now accepted as a junior synonym of *Cirolana*.

Diagnoses to the genus have been given by Bruce (1986), Brusca et al. (1995) and Kensley and Schotte (1989).

### Cirolana songkhla sp. n.

http://zoobank.org/40967001-288A-433E-8B14-A411034CE8A7

http://species-id.net/wiki/Cirolana_songkhla

**Figure 2.**
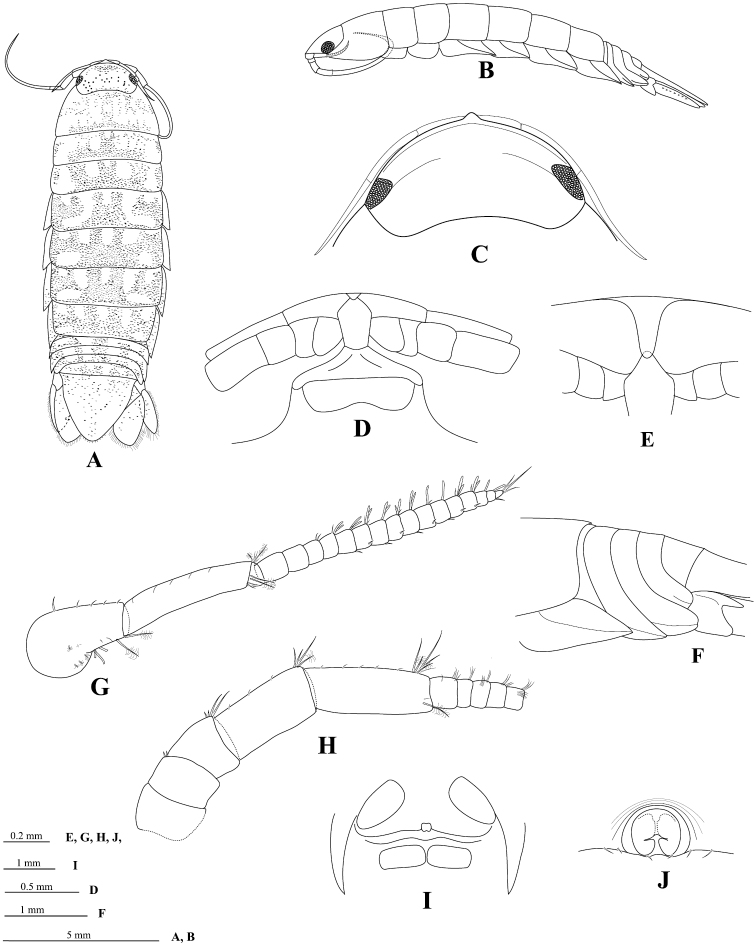
*Cirolana songkhla* sp. n., male holotype (PSUZC-CR0281-01) (13.7 mm) (**A–F**), male paratype (PSUZC-CR0281-2) (11.2 mm) (**G–H**), male paratype (PSUZC-CR0281-2) (13.8 mm) (**I–J**). **A** dorsal view **B** lateral view **C** head, dorsal view **D** frons **E** detail of frontal lamina **F** pleon **G** antennule **H** antennal peduncle **I** antero-ventral view of penial opening **J** ventral view of penial opening.

Figs 2 [Fig F3] [Fig F4] [Fig F5] –6

Cirolana parva ? : Chilton 1924: 883, 1926: 180 (misidentification as *Cirolana parva* Hansen, 1890 in part of freshwater records only; same species as *Cirolana songkhla* sp. n.).

#### Material examined.

Holotype, ♂ (13.7 mm), middle part of Songkhla Lake, Phattalung province, 07°29.09'N, 100°20.11'E, 23 October 2011, gravel bottom and associated with water plants, salinity 4 ppt, coll. E. Rodcharoen (PSUZC-CR0281-01).

Paratypes, 10 ♂ (13.8, 11.2, 12.4, 10.2, 10.3, 10.0, 13.8, 11.8, 11.4, 10.6 mm [dissected]), 1 ♀ (ovig. 8.7 mm [dissected]), further specimens unmeasured, same data as holotype (PSUZC-CR0281-02; MTQ W34265). 8 ♂ (11.1, 12.8, 12.2, 13.9, 13.2, 12.4, 12.2, 9.8 mm [dissected]), 4 ♀ (ovig. 8.6, 8.7 [dissected], 8.8, 9.0 mm), further specimens unmeasured, Klong Ban Klang, Thale Noi, Phattalung province, 07°46.44'N, 100°09.27'E, 27 May 2013 clay bottom, salinity 0.6 ppt, coll. E. Rodcharoen (PSUZC-CR0281-03; MTQ W34266).

#### Additional material.

12 ♂ (unmeasured), same data as holotype (PSUZC-CR0281-04), 38 ♂ and 3 ♀ (unmeasured), same data as paratype PSUZC-CR0281-03; MTQ W34266 (PSUZC-CR0281-05; MTQ W34267), 1 adult ♂ and 3 juvenile (unmeasured), Pak Nam Tapi (estuary), Surat Thani province, 09°10.31'N, 99°21.36'E, 30 October 2012 coll. E. Rodcharoen (PSUZC-CR0281-06).

#### Description of male.

*Body* 3.2 times as long as greatest width, dorsal surfaces smooth, widest at pereonite 5 and pereonite 6, lateral margins subparallel (Figure 2A). *Rostral point* (Figure 2C) present, folded ventrally and posteriorly, in contact with frontal lamina (Figure 2D, E). *Eye* colour dark brown; eyes separated by about 81% width of head (Figure 2C). *Pereonite 1 and coxae* 2–3 each with posteroventral angle rounded; coxae 5–7 with entire oblique carina; posterior margins of pereonites 5–7 smooth (Figure 2B). *Pleon* (Figure 2F) with pleonite 1 largely concealed by pereonite 7; pleonites 3–5 posterior margins smooth; posterolateral angles of pleonite 2 forming acute point, extending posteriorly to anterior of pleonite 4; pleonite 3 with posterolateral margins extending clearly beyond posterior margins of pleonites 4 and 5, narrowly rounded; posterolateral margin of pleonite 4 rounded, clearly extending beyond posterior margin of pleonite 5. *Pleotelson* (Figure 6E) 1.0 times as long as anterior width, dorsal surface without longitudinal carina; lateral margins convex; margins weakly serrate, posterior margin converging to small distinct caudomedial point, with 12 RS (Figure 6E, F).

*Antennule* (Figure 2G) peduncle articles 1 and 2 entirely fused; articles 3 and 4 1.3 times as long as combined lengths of articles 1 and 2, article 3 5.0 times as long as wide; flagellum with 16 articles, extending to middle of pereonite 1. *Antenna* (Figure 2H) peduncle article 4 1.8 times as long as wide, 2.2 times as long as article 3, anterodistal angle with 3 short simple setae and 1 plumose seta; article 5 1.2 times as long as article 4, 2.4 times as long as wide, anterodistal angle with cluster of 4 short simple setae and 2 plumose setae; flagellum with 34 articles, extending to posterior of pereonite 4.

*Frontal lamina* (Figure 2D, E) pentagonal, 2.2 times as long as posterior width; 1.6 times as long as greatest width, lateral margins straight, diverging slightly towards anterior, anterior margin acute, with small median point.

**Figure 3. F3:**
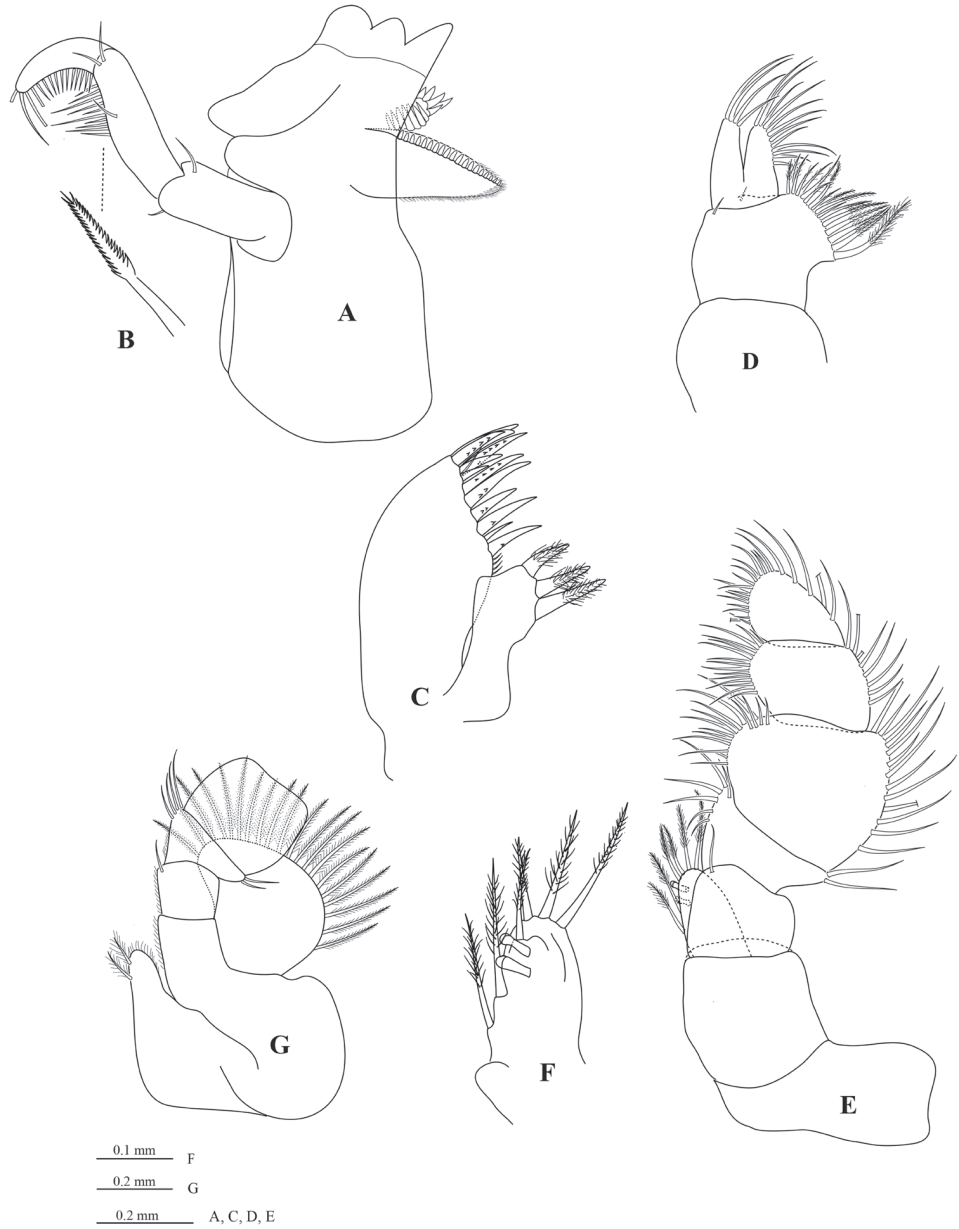
*Cirolana songkhla* sp. n., male paratype (PSUZC-CR0281-2) (11.2 mm) (**A–F**), ovigerous female paratype (PSUZC-CR0281-2) (8.7 mm) (**G**). **A** right madible **B** robust setae **C** right maxillule **D** right maxilla **E** right maxilliped **F** maxilliped endite **G** left maxiliped, basal articles.

*Mandible* molar process (Figure 3A) anterior margin with 19 flat teeth; with proximal cluster of long simple setae; right mandible spine row composed of 13 spines; palp article 2 with 21 distolateral setae, palp article 3 with 22 biserrate RS (Fig. 3B). *Maxillule* (Figure 3C) mesial lobe with 3 large circumplumose RS; lateral lobe with 13 RS (weakly serrated). *Maxilla* (Figure 3D) lateral lobe with 4 long setae; middle lobe with 12 long setae; mesial lobe with 4 distal plumose setae and 14 proximal plumose setae. *Maxilliped palp* (Figure 3E) article 1 mesial margin with 1 slender seta; article 2 mesial margin with 6 slender setae, lateral margin distally with 2 slender setae; article 3 mesial margin with 15 slender setae, lateral margin with 13 slender setae; article 4 mesial margin with 17 slender setae, lateral margin with 9 slender setae; article 5 distal margin with 18 setae, lateral margin with 6 setae; endite (Figure 3F) with 6 long CPS, and 2 coupling setae.

**Figure 4. F4:**
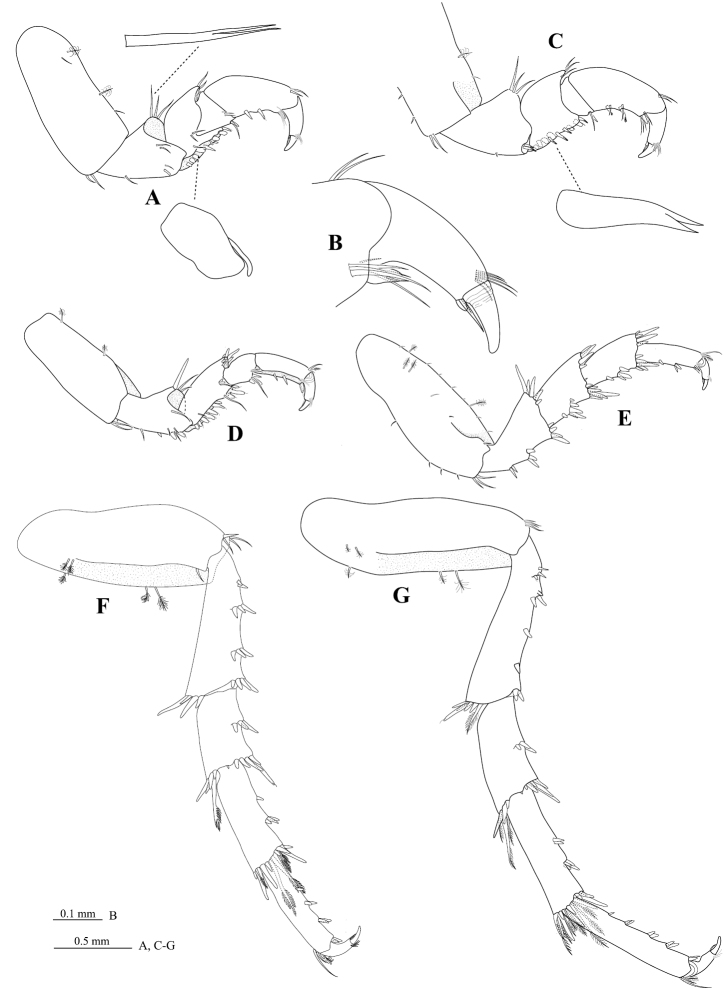
*Cirolana songkhla* sp. n., male paratype (PSUZC-CR0281-2) (11.2 mm). **A** pereopod 1 **B** dactylus of pereopod 1 **C** mesial view, pereopod 1 **D** pereopod 2 **E** pereopod 4 **F** pereopod 6 **G** pereopod 7.

*Pereopod 1* (Figure 4A, C) *basis* 2.3 times as long as greatest width, inferior distal angle with cluster of 2 acute setae; *ischium* 0.5 times as long as basis, inferior margin with 1 acute seta, inferior distolateral margin with 2 setae (1 molariform RS and 1 acute seta), median distolateral margin with 2 acute setae, superior distal margin with 3 RS; *merus* inferior margin with 6 molariform RS, (set in rows of 4 and 2), superior distal angle with 3 setae (slender); *carpus* inferior distal margin with 2 setae (1 RS and 1 acute seta); *propodus* 2.0 times as long as wide, inferior margin with 2 RS; *dactylus* (Figure 4B) 0.5 times as long as propodus. *Pereopod 2* (Figure 4D) *ischium* inferior margin with 2 stout, bluntly rounded RS, superior distal margin with 3 RS; *merus* inferior margin with 10 stout RS, set in one row, superior distal margin with 6 acute RS; *carpus* inferodistal angle with 3 RS (plus 1 slender seta); *propodus* 3.5 times as long as wide; *dactylus* 0.7 times as long as propodus. *Pereopod 3* similar to pereopod 2. *Pereopod 4* (Figure 4E) intermediate in from between pereopod 3 and pereopod 5. *Pereopod 6* (Figure 4F) similar to pereopod 7. *Pereopod 7* (Figure 4G) *basis* 3.0 times as long as greatest width, superior margin weakly convex, inferior margin with 3 palmate setae; *ischium* 0.8 times as long as basis, inferior margin with 8 RS (set in groups of 3, 3, 1 and 1), superior distal angle with 6 RS (5 simple, 1 biserrate), inferior distal angle with 2 RS; *merus* 0.5 times as long as ischium, 2.0 times as long as wide, inferior margin with 3 RS, superior distal angle with 6 RS (3 simple, 3 biserrate), inferior distal angle with 7 RS; *carpus* 0.7 times as long as ischium, 2.4 times as long as wide, inferior margin with 6 RS (set in groups of 2 and 4), superior distal angle with 11 RS (4 simple, 7 biserrate), inferior distal angle with 9 RS (6 simple, 3 biserrate); *propodus* 0.8 times as long as ischium, 5.4 times as long as wide, inferior margin with 7 RS (set in groups of 1, 2, 2 and 2), superior distal angle with 3 slender setae and 1 palm seta, inferior distal angle with 2 RS; *dactylus* 0.3 times as long as propodus.

*Penes* (Figure 2I, J) medially united low papillae.

**Figure 5. F5:**
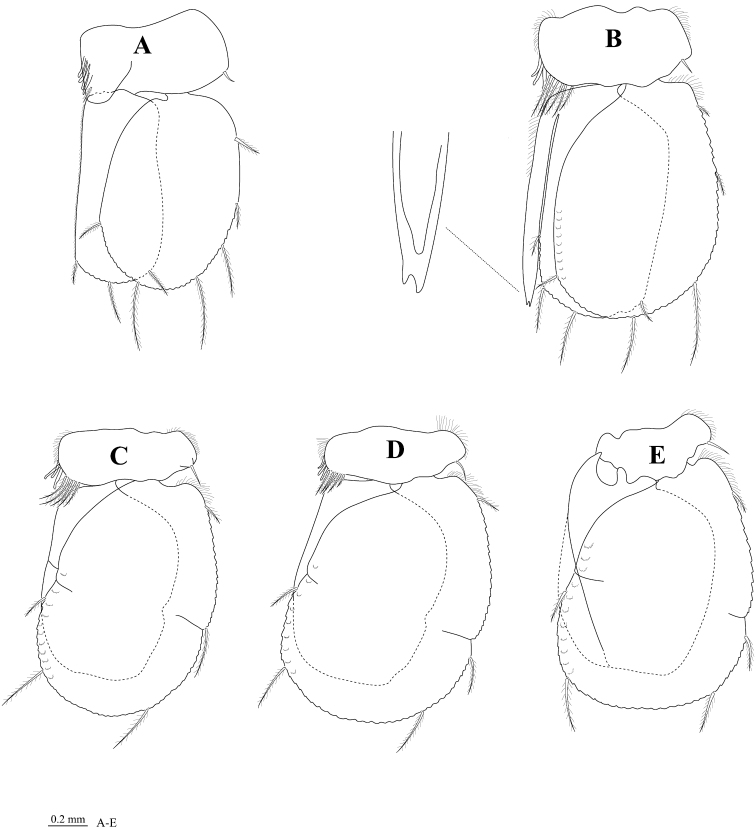
*Cirolana songkhla* sp. n., male paratype (PSUZC-CR0281-2) (11.2 mm) **A–E** pleopods 1–5 respectively.

*Pleopod 1* (Figure 5A) exopod 1.3 times as long as wide, lateral margin weakly convex, distally broadly rounded, mesial margin weakly convex, with ~35 PMS from distal one-third; endopod 2.1 times as long as wide, distally broadly rounded, lateral margin sinuate, with ~16 PMS on distal margin only; peduncle 1.9 times as wide as long, mesial margin with 4 coupling hooks. *Pleopod 2* (Figure 5B) exopod with ~52 PMS, endopod with ~24 PMS; *appendix masculina* with parallel margins, 1.0 times as long as endopod, distally notch. *Pleopod 3* (Figure 5C) endopod without PMS, exopod with ~60 PMS. *Pleopod 4* (Figure 5D) endopod without PMS, exopod with ~60 PMS. *Pleopod 5* (Figure 5E) endopod without PSM, exopod with ~54 PMS. Pleopods 2–5 peduncle distolateral margin with prominent acute RS, 3–5 endopods with distomesial serrate scales.

**Figure 6.**
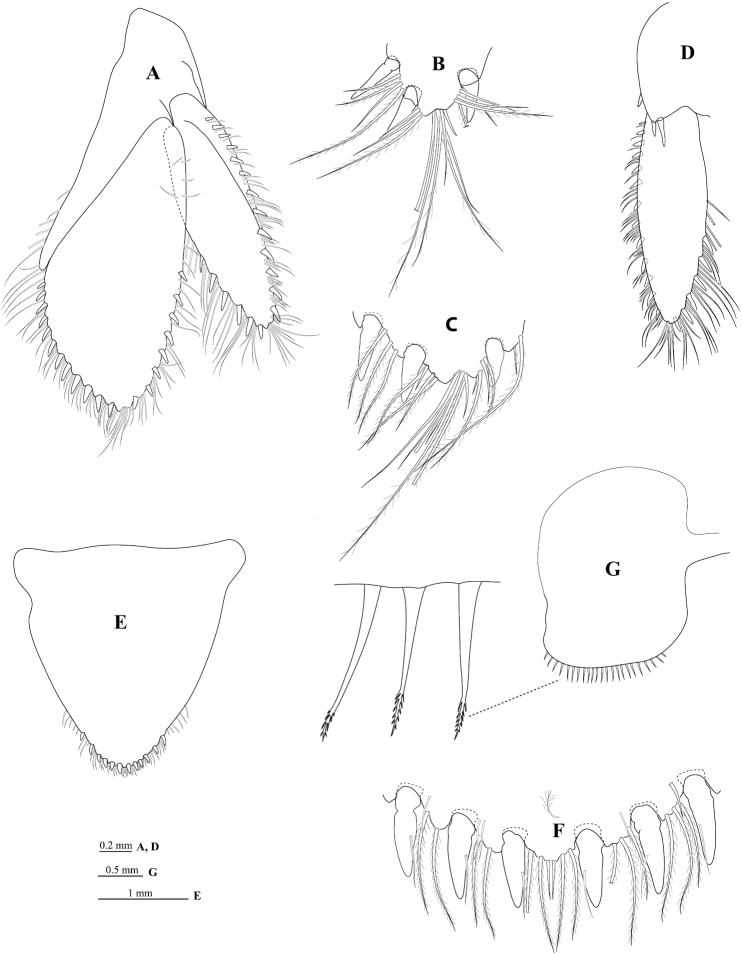
*Cirolana songkhla* sp. n., male paratype (PSUZC-CR0281-2) (11.2 mm) (**A–F**), ovigerous female (PSUZC-CR0281-2) (8.7 mm) (**G**). **A** uropod **B** uropod exopod apex **C** uropod endopod apex **D** uropod exopod **E** pleotelson **F** detail of pleotelson apex **G** oostegite 4.

*Uropod* (Figure 6A) peduncle ventrolateral margin (Figure 6D) with 2 RS, lateral margin with 1 mesial short acute RS, posterior lobe about one-half as long as endopod; rami extending beyond pleotelson, marginal setae in single tiers, apices acute. *Endopod* (Figure 6A) apically sub-bifid, medial process prominent (Figure 6C); lateral margin weakly convex, with 8 RS, mesial margin strongly convex, with 12 RS. *Exopod* (Figure 6A) not extending to end of endopod, 3.3 times as long as greatest width, apically sub-bifid, medial process prominent (Figure 6B); lateral margin weakly convex, with 13 RS; mesial margin weakly convex, with 6 RS.

#### Female.

Similar to male but on average smaller. Antennal flagellum slightly longer, extending to anterior of pereonite 5; maxilliped with lamina vibrans (Figure 3G); brood pouch composed of 5 pairs of oostegites (Figure 6G) arising on sternites 1–5 (as recorded for some other *Cirolana* species, such as *Cirolana (Anopsilana) barnardi* (Bruce, 1992) and *Cirolana kokoru* Bruce, 2004), lateral margin of oostegite 4 with ~23 slender setae.

#### Variation.

Pleotelson (n=23 [18♂ and 5♀]) with 12–15 RS, with 14 RS (7+7) most frequent (74%), 12 (4%) and 15 (4%) occurring only once. Uropod endopod mesial margin with 11–13 RS, with 12 (52%) and 11(39%) most frequent, lateral margin with 8–10 RS, with 8 (52%) and 9 (39%) most frequent; exopod mesial margin with 5–8 RS, with 7 (39%) and 6 (35%) most frequent, lateral margin with 12–14, with 13 (74%) most frequent.

#### Size.

Adult males (n=19) 9.8–13.9 mm (mean 11.9 mm); ovigerous females (n=5) 8.6–9.0 mm (mean 8.8 mm).

#### Remarks.

The presence of abundant chromatophores and lack of ornamentation on the posterior pereonites, pleonites and pleotelson excludes *Cirolana songkhla* sp. n. from the *Cirolana* ‘tuberculate-group’ (see Bruce 1986). Although *Cirolana songkhla* sp. n., with smooth dorsal surface, seems to belong to the *Cirolana* ‘*parva*-group’ of Bruce (2004), there are several characters that differ to that group. The body size of *Cirolana songkhla* sp. n. is significantly larger than most tropical ‘*parva*-group’ species, most which do not exceed 7 mm. Moreover, the uropods and pleotelson margins are far more heavily armed with robust setae than the ‘*parva*-group’; and the coxae of *Cirolana songkhla* sp. n. are more visible in dorsal view than usually for the ‘*parva*-group’.

*Cirolana songkhla* sp. n. is characterized by lacking plumose setae on endopods of pleopods 3–5. This character is particularly associated with brackish and freshwater cirolanid species, formerly placed in the genus and then subgenus *Anopsilana* (Bruce 1981, Botosaneanu and Iliffe 1997). However, *Cirolana songkhla* sp. n. can be distinguished from the other species of *Cirolana (Anopsilana)* by having well-developed eyes (absent in freshwater cave species) and smooth dorsal surfaces [nodular and tubercular in species, such as *Cirolana fluviatilis* Stebbing, 1902 and *Cirolana willeyi* Stebbing, 1904].

Only *Cirolana barnardi* (Bruce, 1992) from tropical eastern Australia is similar to *Cirolana songkhla* sp. n. having in common a smooth dorsal surface, rostral point and pentagonal frontal lamina. However, the two species can be clearly distinguished by body size of *Cirolana songkhla* sp. n., which is larger than that of *Cirolana barnardi* (male average at 11.9 VS 3.9 mm, ovigerous female at 8.8 VS 4.0 mm). Furthermore, *Cirolana songkhla* sp. n. has more numerous robust setae on the uropodal rami and pleotelson margin than *Cirolana barnardi*; uropod exopod lateral margin with 12–14 RS (VS 7–10), mesial margin with 5–8 RS (VS 5–6); endopod lateral margin with 8–10 RS (VS 3–4), mesial margin with 11–13 RS (VS 5–7) and pleotelson, posterior margin converging to small distinct caudomedial point (VS posterior margin subtruncate), with 12–15 RS (VS 7–10).

*Cirolana parva* Hansen, 1890 has been recorded in freshwater from Thailand by Chilton (1924, 1926) from Songkhla Lake and Talé Sap. *Cirolana parva* is, unequivocally, restricted to marine habitats in Central America and the Caribbean (Bruce and Bowman 1982), with all Indo-Pacific records being misidentifications (see also Bruce 1986, 1995, 2004; Schotte and Kensley 2005). It is probable that Chilton’s specimens, misidentified as *Cirolana parva*, are the same species as *Cirolana songkhla* sp. n. Chilton (1924) gives a figure (figure 5, but the locality of the specimens is not stated) of the pleotelson and uropods of the Songkhla Lake species, which has a more acute pleotelson apex but is otherwise compatible with the present material and records the size of the Talé Sap specimens as 9 mm, also compatible with the present species. For those reasons, we provisionally include these records from freshwater in the synonymy for *Cirolana songkhla* sp. n. Chilton (1924) also included several marine localities and some uncertain localities, and these other records are regarded as being of unknown identity.

#### Etymology.

*Cirolana songkhla* sp. n. is named for the type locality.

### Key to brackish species of *Cirolana* in Thailand

**Table:** 

1	Anterior margin of head without rostral point; frontal lamina anterior margin rounded	*Cirolana fluviatilis*
–	Anterior margin of head with rostral point, folded ventrally and posteriorly, in contact with frontal lamina; frontal lamina pentagonal	2
2	Body dorsal surface without ornamentation; pleotelson lateral margins convex	*Cirolana songkhla* sp. n.
–	Body dorsal surface with tubercles on pereonites, pleonites and pleotelson; pleotelson lateral margins concave	*Cirolana willeyi*

## Supplementary Material

XML Treatment for Cirolana

XML Treatment for Cirolana songkhla
